# Design and Pre-Clinical Evaluation of a Universal HIV-1 Vaccine

**DOI:** 10.1371/journal.pone.0000984

**Published:** 2007-10-03

**Authors:** Sven Létourneau, Eung-Jun Im, Tumelo Mashishi, Choechoe Brereton, Anne Bridgeman, Hongbing Yang, Lucy Dorrell, Tao Dong, Bette Korber, Andrew J. McMichael, Tomáš Hanke

**Affiliations:** 1 Medical Research Council Human Immunology Unit, Weatherall Institute of Molecular Medicine, University of Oxford, The John Radcliffe, Oxford, United Kingdom; 2 Los Alamo National Laboratory, Theoretical Biology and Biophysics, Los Alamos, New Mexico, United States of America; 3 The Santa Fe Institute, Santa Fe, New Mexico, United States of America; University of California at San Francisco, United States of America

## Abstract

**Background:**

One of the big roadblocks in development of HIV-1/AIDS vaccines is the enormous diversity of HIV-1, which could limit the value of any HIV-1 vaccine candidate currently under test.

**Methodology and Findings:**

To address the HIV-1 variation, we designed a novel T cell immunogen, designated HIV_CONSV_, by assembling the 14 most conserved regions of the HIV-1 proteome into one chimaeric protein. Each segment is a consensus sequence from one of the four major HIV-1 clades A, B, C and D, which alternate to ensure equal clade coverage. The gene coding for the HIV_CONSV_ protein was inserted into the three most studied vaccine vectors, plasmid DNA, human adenovirus serotype 5 and modified vaccine virus Ankara (MVA), and induced HIV-1-specific T cell responses in mice. We also demonstrated that these conserved regions prime CD8^+^ and CD4^+^ T cell to highly conserved epitopes in humans and that these epitopes, although usually subdominant, generate memory T cells in patients during natural HIV-1 infection.

**Significance:**

Therefore, this vaccine approach provides an attractive and testable alternative for overcoming the HIV-1 variability, while focusing T cell responses on regions of the virus that are less likely to mutate and escape. Furthermore, this approach has merit in the simplicity of design and delivery, requiring only a single immunogen to provide extensive coverage of global HIV-1 population diversity.

## Introduction

Despite twenty-five years of global effort, an effective vaccine against the human immunodeficiency virus type 1 (HIV-1) remains elusive. Induction of broadly neutralizing antibodies against HIV-1 is very difficult, yet it is the key to all other protective anti-viral vaccines[1]. Therefore consideration of HIV-1 vaccine candidates that stimulate cellular immunity has been the focus of many recent vaccines[2]. Although recent advances in vector design have generated optimism in this field[3], [4], these technologies still need to address the extreme variability of HIV-1, whereby co-circulating viruses may differ in over 20% of their proteome[5], [6]. Thus, while novel vectors and heterologous prime-boost combinations are getting better at inducing higher frequencies of HIV-1-specific T cells, less attention has been paid to how these vaccines can elicit T cells capable of recognizing multiple HIV-1 variants.

There are several approaches for dealing with the HIV-1 diversity. One optimistic view is that a single clade may induce sufficiently cross-reactive T-cell responses to protect against other variants of both the same and heterologous clades. The choice of a natural isolate can be based on having the closest sequence to all others, or picking a strain derived from acute infection and arguing that there is a convergence of viral sequences during transmission[7]. However, even if a single variant elicits responses that confer some cross-reactive protection, such protection is likely to be only partial and thus it is well worth attempting to design vaccine immunogens with enhanced cross-reactive potential. Although there are numerous reports of cross-clade reactive HIV-1-specific CD8^+^ T cell responses[8]–[12], use of unphysiologically high concentrations of variant peptides make the biological relevance of many of these results uncertain. In contrast, there are ample examples of highly specific T cell receptors sensitive to single amino acid (aa) changes[13]–[19], as well as compelling evidence of HIV-1 variants escaping existing T cell responses in infected individuals by single mutations in epitopes[2], [20]–[22]. In vitro, systematic studies employing all possible single aa substitutions in each position of an MHC class I epitope indicated that as few as one third of such epitope variants were recognized by a given T cell receptor[13], [19]. These results are in agreement with theoretical predictions proposed for cross-recognition of MHC class I-presented peptides by T cell receptors[23]. Thus the use of a single natural isolate for a vaccine has a high risk of not protecting against a different clade, nor against many variants of the same clade.

A second approach to HIV-1 diversity derives vaccine immunogens from ‘centralized’ sequences, which employ consensus/average, or centre-of-the-tree[24] sequences or extrapolated aa to a common clade or group ancestor[5]. Centralized sequences are designed to minimize the sequence differences between a vaccine immunogen and circulating viruses[5], [24]–[26]. So far they have proven immunogenic and able to elicit T cell responses in small animal studies[24], [27]–[29] and clinical trials[30]–[32], providing experimental support for their further development. Early results for centralized immunogens for the entire group M are promising in that initial immunogenicity studies in mice yielded T-cell responses that were comparable to within-clade responses for many clades[29], however, this strategy may be stretched too far for optimal coverage of CD8^+^ T cell epitope variants of the whole group M[18], [19], [33].

In a third approach, vaccines deliver a cocktail of immunogens derived from different clades[34]–[36]. While initial results have been encouraging and responses to each antigen in the cocktail were observed[36], attention still needs be paid to possible immune interference, such as epitope antagonism, between different, but closely related peptide sequences in the vaccine, which may be limiting responses to some epitopes. Antagonism of T cell responses by altered epitope peptide ligands has been demonstrated both *in vitro* [37], [38] and *in vivo* [18], [39]–[42]. It can occur when a host capable of mounting a response to an agonist epitope is simultaneously exposed to an antagonist epitope variant, which interferes with the induction of the T cell response to the agonist epitope and leads to a defective response. Thus, the breadth of responses induced by cocktail approaches should be carefully monitored when such vaccines are used[18].

A fourth approach uses computational methods for assembling a polyvalent vaccine candidate that optimize the coverage of T cell epitopes. ‘Mosaic’ immunogens[43] are based on intact proteins and retain the probability for natural processing and presentation of T cell epitopes. Their potential problems are similar to those of other cocktails of natural proteins, i.e. immune interference and inclusion of both variable and conserved regions, whereby responses to variable regions may draw attention away from potentially more useful conserved targets. The impact of these processes will be only resolved in vaccine studies. An alternative means of designing immunogens contending with the HIV-1 variation is the COT+ method[44], which combines a central sequence with a set protein fragments designed to help cover diversity.

Here, we describe a further alternative that may have considerable advantages. We describe the construction and experimental testing in mice and humans of a novel multi-clade immunogen derived only from highly conserved regions of the HIV-1 consensus proteome, which was designed to provide extensive coverage of the principle HIV-1 clades A, B, C and D, while minimizing the possible occurrence of the epitope interference. It has the potential considerable advantage of focusing the T cell responses on the most conserved parts of the virus and thus overcoming the usual patterns of immunodominance, while making it difficult for the virus to escape without a likely significant cost to its fitness. This approach has also merit in the simplicity of design and delivery, requiring only a single immunogen to provide extensive coverage of global HIV-1 population diversity.

## Methods

### Preparation of the pTH.HIV_CONSV_ and pTH.HIV_CONSV_dH DNA vaccines

The synthetic gene coding for HIV_CONSV_ (GeneArt) was subcloned into plasmid pTH [45] and the codons coding for the H epitope were deleted using a PCR assembly to generate pTH.HIV_CONSV_dH. The plasmid DNA for immunizations was prepared using the Endo-Free Gigaprep (Qiagen) and stored at −20°C until use.

### Preparation of the AdHu5.HIV_CONSV_ and AdHu5.HIV_CONSV_dH vaccines

Recombinant adenoviruses were obtained using the AdEasy™ Adenoviral Vector System (Stratagene), following the manufacturer's instructions. Both adenoviruses expressed the green fluorescent protein as a marker.

### Preparation of the MVA.HIV_CONSV_ and MVA.HIV_CONSV_dH vaccines

Recombinant MVAs (rMVAs) were made as described previously [46]. Briefly, chicken embryo fibroblast (CEF) cells grown in Dulbeco's Modified Eagle's Medium supplemented with 10% FBS, penicillin/streptomycin and glutamine (DMEM 10) were infected with parental MVA at MOI 1 and transfected using Superfectin (Qiagen) with 3 µg of pSC11.HIV_CONSV_ or pSC11.HIV_CONSV_dH carrying also the β-galactosidase gene as a marker. Two days later, the total virus was harvested and used to re-infect CEF cells. rMVAs were subjected to five rounds of plaque purification, after which a master virus stock was grown, purified on a 36% sucrose cushion, titered and stored at –80°C until use.

### Immunofluorescence

Six-well tissue culture plates containing sterile microscope coverslips treated with 0.01% poly-L-lysine (Sigma) were seeded with 1×10^5^ cells/well of HEK293T. After 12–24 h, cells reached 80% confluency and were transfected with 5 µg of a selected plasmid using the Superfectin (Qiagen), according to the manufacturer's recommendations, or infected with a selected recombinant viral vector at MOI 1 or 10. Cells were then incubated for 18–24 h, fixed with 0.5% formaldehyde, washed with PBS, their membranes perforated with 50% ethanol for 5 min, 70% ethanol for 10 min, and 50% ethanol for 3 min. Cells were washed again and blocked overnight with 2% FCS in PBS (FCS/PBS) at 4°C. The FCS/PBS solution was replaced by a 1/200 working dilution of a primary mouse monoclonal antibody against the Pk tag (Serotec) in FCS/PBS and incubated for 3–18 h at 4°C. Cells were subsequently washed three times with PBS, and incubated with a 1/500 dilution of the Alexa fluor 594-conjugated secondary chicken anti-mouse monoclonal antibody (Molecular Probes) in FCS/PBS for 2 h at room temperature or for 3–18 h at 4°C. Alternatively, a FITC-conjugated mouse monoclonal antibody against the Pk tag was used at a 1/500 dilution in FCS/PBS, and incubated for 2 h at room temperature or 3–18 h at 4°C. The cells were then washed three times with PBS, mounted on a microscope slide with Vectashield DAPI nuclear stain (Vector Laboratories) and photographed on a Zeiss fluorescence microscope.

### Mice

For all animal experiments, groups of four 5- to 8-week-old female BALB/c or HHD mice were used. All animal procedures and care were approved by a local Ethical Committee and strictly conformed to the UK Home Office Guidelines.

### Mouse immunizations and preparation of splenocytes

Under general anaesthesia, mice were immunized i.m. with 100 µg of pTH.HIV_CONSV_ DNA, or 10^6^ PFU of AdHu5.HIV_CONSV_ or 10^6^ PFU of MVA.HIV_CONSV_. On the day of sacrifice, spleens were removed and pressed individually through a cell strainer (Falcon) using a 5-ml syringe rubber plunger. Following the removal of rbc with Rbc Lysis Buffer (Sigma), splenocytes were washed and resuspended in Lymphocyte Medium [R-10 (RPMI 1640 supplemented with 10% FCS, penicillin/ streptomycin), 20 mM HEPES and 15 mM 2-mercaptoethanol] at concentration of 2×10^7^ cells/ml.

### Peptides and peptide pools

HPLC-purified, overlapping 15- by 11-mer peptides spanning the entire HIV_CONSV_ protein were obtained from Sigma Genosys. Peptides were at least 80% pure as verified by mass spectrometry. Individual peptides corresponding to epitopes were synthesised in an in-house facility (Weatherall Institute of Molecular Medicine, Oxford). All peptides were dissolved in DMSO (Sigma) at a concentration of 50 mg/ml, and stored at −80°C.

### Murine intracellular cytokine staining

Two million splenocytes were added to each well of a 96-well round-bottomed plate (Falcon) and pulsed with peptides or peptide pools and kept at 37°C, 5% CO_2_ for 90 min, followed by the addition of GolgiStop™ (BD Biosciences). After a further 5-h incubation, reaction was terminated, the cells washed with FACS wash buffer (PBS, 2% FCS, 0.01% Azide) and blocked with anti-CD16/32 (BD Biosciences) at 4°C for 30 min. All subsequent antibody stains were performed using the same conditions. Cells were then washed and stained with anti-CD8-PerCP or anti-CD4-PerCP (BD Biosciences), washed again and permeablized using the Cytofix/Cytoperm™ kit (BD Biosciences). Perm/Wash buffer (BD Biosciences) was used to wash cells before staining with anti-IL-2-FITC, anti-TNF-α-PE and anti-IFN-γ-APC (BD Biosciences). Cells were fixed with CellFIX™ (BD) and stored at 4°C until analysis.

### Murine ELISPOT assay

The ELISPOT assay was performed using the Becton Dickinson IFN-γ ELISPOT kit according to the manufacturer's instructions. The membranes of the ELISPOT plates (BD Immunospot™ ELISPOT Plates) were coated with purified anti-mouse IFN-γ antibody diluted in PBS to a final concentration of 5 µg/ml at 4 °C overnight, washed once in R-10, and blocked for 2 h with R-10. A total of 2.5×10^5^ splenocytes were added to each well, stimulated with or without peptide for 16 h at 37°C, 5% CO_2_ and lysed by incubating 2x with deionized water for 5 min. Wells were then washed 3x with PBS 0.05% Tween-20, incubated for 2 h with a biotinylated anti-IFN-γ antibody diluted in PBS 2% FCS to a final concentration of 2 µg/ml, washed 3x in PBS 0.005% Tween-20 and incubated with 50 mg/ml horseradish peroxidase-conjugated to avidin in PBS 2% FCS. Wells were washed 4x with PBS 0.005% Tween-20 and 2x with PBS before incubating with an AEC substrate solution [3-amino-9-ethyl-carbazole (Sigma) dissolved at 10 mg/ml in Dimethyl formaldehyde and diluted to 0.333 mg/ml in 0.1 M acetate solution (148 ml 0.2 M acetic acid and 352 ml 0.2 M sodium acetate in 1 liter pH 5.0) with 0.005% H_2_O_2_]. After 5–10 min, the plates were washed with tap water, dried and the resulting spots counted using an ELISPOT reader (Autoimmune Diagnostika GmbH).

### ^51^Chromium-release assay

Isolated mouse splenocytes were restimulated *in vitro* with 2 µg/ml of peptide in the Lymphocyte Medium for 5 days, at 37°C 5% CO_2_. On day 5 the cells were washed three times in R-0 and diluted two-fold in a 96-well U-bottom plate (Nunc) to yield the different effector to target ratios. ELA A2-K^d^ or JK A2-K^d^ target cells were labelled with ^51^Chromium at 37°C 5% CO_2_ for 90 min with or without appropriate peptide, washed three times with R-0, added to the effector cells at 5×10^3^ target cells per well, and incubated at 37°C 5% CO_2_ for 4–6 h. The percentage of peptide-specific lysis was calculated as [(sample release-spontaneous release)/(total release-spontaneous release)]×100. The spontaneous release was less than 10% of the maximum release.

### Human PBMC samples

For healthy lab subjects, PBMC separation was performed within 2 h of blood receipt. Blood was layered onto Ficoll (Sigma- Aldrich) and centrifuged (40 min, 400 *g*, without brake) at room temperature. Following centrifugation, the cellular interface was removed, diluted in Hanks buffer (Sigma-Aldrich), and re-centrifuged. Cryopreserved PBMC samples from vaccine clinical trial participants in Oxford and African patients were used for detection of HIV-1-specific effectors. Cells were washed once more with 50 ml RPMI (Sigma-Aldrich) and then suspended in 10 ml RPMI for counting. Cells were counted using a Coulter Z1 Counter (Beckman-Coulter). Trypan blue exclusion (Sigma-Aldrich) was used to estimate the percentage of viable cells. All studies involving human samples were approved by local Ethical Review Panels and all patients gave an informed consent for donation of blood samples.

### Short-term culture of PBMC

Short-term cell lines were set up as described previously[31]. Briefly, on day 0, fresh or frozen PBMCs were washed three times in R-0 and resuspended at 2×10^6^ cells/ml in RAB-10 (RPMI 1640, 10% human AB serum) supplemented with 25 ng/ml of IL-7 (R&D Systems). Twelve-well tissue culture plates (Nunc) were seeded with 0.9 ml of the PBMC suspension. Hundred ml of either Pool 1-3, Pool 4-6, or the FEC Pool (positive control of Flu-EBV-CMV CD8 epitopes) was added to each well for a final concentration of 1.5 µg/ml for each individual peptide. On day 3 and 7, 1800 IU of IL-2 (Chiron) were added to each well, as well as 1 ml of RAB-10 on day 7. On day 10, cells are washed twice in PBS, resuspended in fresh RAB-10 and rested for 26 to 30 h. Cells were subsequently tested for IFN-γ production in an ELISPOT assay with or without CD8^+^ cell depletion as described below.

### CD8^+^ cell depletion

CD8^+^ cells were depleted using the Dynabead Depletion Kit (Dynal) following the manufacturer's recommendation. Briefly, PBMCs were resuspended in a small volume with biotinylated anti-CD8 monoclonal antibody (BD Biosciences), incubated for 20 min at 4°C, the excess antibody was then washed off, cells were incubated with Dynabeads for 20 min at room temperature on an orbital shaker, and separated by a magnet. The depleted cells were collected and washed twice.

### IFN-γ ELISPOT assay

PVDF membrane ELISPOT 96-well plates (Nunc) were pre-wet by dispensing 50 µl of 70% ethanol, incubating at room temperature for 5 min and washing three times with endotoxin free PBS (Sigma). Plates were then coated overnight at 4°C with 50 µl of a 10 µg/ml purified anti-human IFN-γ antibody solution in PBS. Plates were blocked with R-10 for 2 h, 80,000 cells/wells in 50 µl of R-10 were added to each well (40,000 cells for FEC lines), as well as the appropriate peptide pool in 50 µl of R-0 (final concentration of 1.5 µg/ml or each individual peptide) or relevant controls from pre-aliquoted peptide plates. Plates were incubated at 37°C, 5% CO_2_ for 14 to 18 h. Wells were then washed six times with PBS 0.05% tween-20 and incubated for 2 h with a biotinylated anti-IFN-γ antibody diluted in PBS 0.5% BSA to a final concentration of 1 µg/ml. Wells were washed again six times with PBS 0.05% tween-20 and incubated for 1 h with a horseradish peroxidase complex (Vector Laboratories) in PBS. Wells were washed four times with PBS 0.05% tween-20, two times with PBS, and incubated for 4 min with an AEC substrate solution (3-amino-9-ethyl-carbazole (Sigma) dissolved at 10 mg/ml in dimethyl formaldehyde and diluted to 0.333 mg/ml in 0.1 M acetate buffer [148 ml 0.2 M acetic acid and 352 ml 0.2 M sodium acetate in 1l of distilled water, pH 5] with 0.005% H_2_O_2_). Wells were finally washed three to five times with tap water to stop the reaction, dried, and spots were counted on an ELISPOT reader (Autoimmune Diagnostika). Results were expressed as Spot Forming Units per million cells (SFU/10^6^ cells). Responses were considered positive if they were four times higher than background (no peptide) and if the background was less than 100 SFU/10^6^ cells.

### Statistical analysis

An unpaired student t-test was used to determine significant difference between the averages of mock-stimulated splenocytes and splenocytes restimulated with a particular peptide in ICS assays in BALB/c mice, and between the averages of mock-stimulated splenocytes and splenocytes restimulated with a particular peptide pool in IFN-γ ELISPOT assays in HHD mice, PBMC unpulsed and peptide-pulsed, and was performed using the program available at http://www.physics.csbsju.edu/stats/t-test.html. Responses were defined as positive if p<0.05.

## Results

### Design of the HIV_CONSV_ immunogen

**Figure 1:**
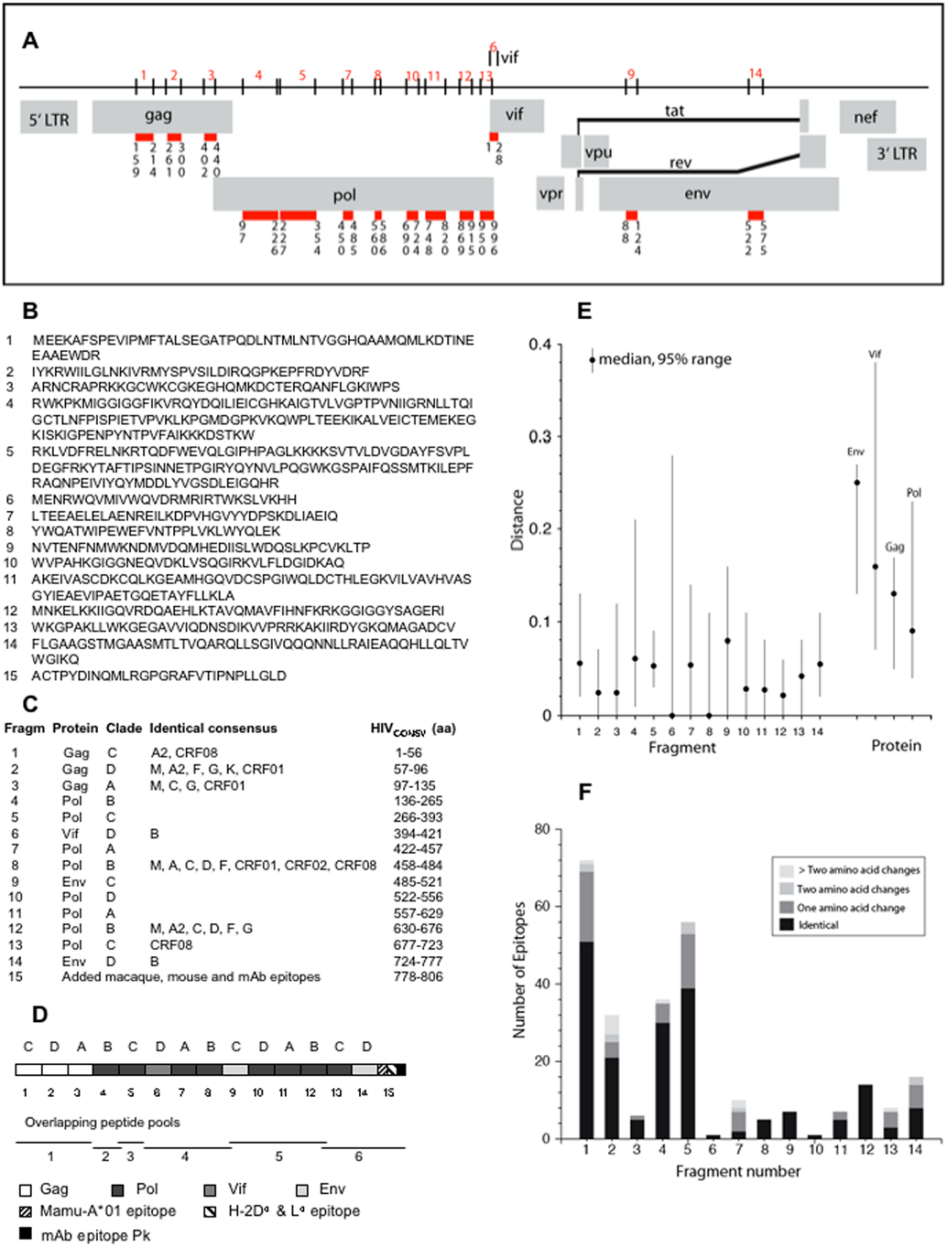
The HIV_CONSV_ immunogen. (A) Localization of the 14 most highly conserved regions of the HIV-1 proteome. The numbers written vertically under each fragment boundary indicate the first and last aa positions using the HXB2 reference strain numbering (http://www.hiv.lanl.gov/content/hiv-db/LOCATE/locate.html). (B) Predicted aa sequence of the the HIV_CONSV_ immunogen with indicated fragment numbers. (C) Summary of the fragments including: the fragment number; the protein in which it was embedded; the clade of the consensus sequence selected for inclusion in the immunogen, alternating between clades A–D; additional clades that have identical HIV_CONSV_; and position numbers in the chimeric vaccine. The number of additional clades with identical consensus sequences to selected clade reflects the high level of conservation in these regions, and is encouraging in terms of the global potential of the vaccine. The consensus sequences compared were to the M group consensus, clades A–K, and three very common recombinant circulating forms CRF01 (common in Asia and Africa), CRF02 (common Africa), CRF08 (common in China) retrieved from the Los Alamos database 2004 consensus alignment (http://www.hiv.lanl.gov/content/hiv-db/CONSENSUS/M_GROUP/Consensus.html). (D) Schematic representation of the HIV_CONSV_ immunogen (not drawn to scale) indicating clade anternation (above), overlapping peptide pool derivation and protein origin by colour coding. (E) Hamming distances between the HIV_CONSV_ antigen fragments and the global circulating viral sequences. The full M group alignment, including recombinant sequences, was used for the comparison. The Los Alamos database alignment contains only one sequence person, and contains sequences from between 600 and 1000 individuals in these proteins. The Hamming distance range for 95% of the sequences relative to the vaccine immunogen is given by the vertical lines. The distances between the full length natural proteins were then calculated relative to HXB2 reference strain Env, Vif, Gag and Pol sequences for comparison. Distance measures are minimal estimates, as gaps inserted in regions where insertions and deletions occur were not counted. (F) Numbers of known CD8^+^ T cell epitopes (defined to within 12 aa or less in the Los Alamos HIV-1 database) in each of the 14 conserved protein fragments included in the HIV_CONSV_ immunogen are shown. When more than one HLA class I presenting molecules can present the same HIV-1 epitope, then each is counted as a distinct epitope; if more than one sequence variant has been described as an epitope presented by the same class I molecule, then these are counted as a distinct epitopes; however, if an HLA serotype and genotype that are potentially the same are each described as presenting the same epitope (like *A2* and A*0201) they are scored as a single epitope.

A novel immunogen, termed HIV_CONSV_ (for conserved), was designed as a chimaeric protein and assembled from the most highly conserved domains among the HIV-1 clade A, B, C and D proteomes. First, a decision was taken that the *HIV_CONSV_* gene should be approximately 2.5 kbp in size, which makes it suitable for most currently used genetic vaccine vectors and is likely to support a high protein expression. Two and a half thousand nucleotides translated into fourteen, 27- to 128-aa-long, most conserved regions of the HIV-1 proteins (Fig. 1A). The centralized sequence method was employed and the HIV_CONSV_ immunogen was assembled from segments derived from one of the four within-clade consensus sequences (Fig. 1B) to reflect the fact that even the most conserved regions of HIV-1 are somewhat variable. It should be noted that because these regions are so highly conserved, often the consensus for one clade perfectly matched the consensus sequences of the other clades or indeed the group M (Fig. 1C), enhancing the potential for eliciting globally relevant cross-reactive responses. To keep the vaccine simple and minimize occurrence of immune interference of T cell responses while ensuring a good coverage of all the four major clades, we alternated the clades of individual segments in the ‘string’ (Fig. 1C and D). Epitopes recognized by rhesus macaque and mouse CD8^+^ T cells, and a mAb[47]–[49] were added to the C-terminus of the HIV_CONSV_ immunogen (Fig. 1B–D), to facilitate the vaccine pre-clinical development.

Alignments of the HIV_CONSV_ immunogen with the global HIV-1 sequences of group M including recombinant forms revealed that at least half of the sequences in the Los Alamos database are identical to fragments 6 and 8 (the median distance = 0), while fragments 2, 3, 10, 11, and 12 differ in less than 3% of their aa positions when compared to half of the sequences (median <0.03). The largest distance from the circulating global sequences displayed fragment 9 with differences in just over 7% aa positions.

Conserved HIV-1 protein regions were included into the HIV_CONSV_ immunogen irrespective of whether of not they contained known T cell epitopes, however, every conserved fragment in the HIV_CONSV_ contains at least one known human epitope (Fig. 1F). In fact, 270 (24%) of the 1112 distinct published CD8^+^ T cell epitopes smaller than 12 aa described in the Los Alamos HIV-1 database are embedded in these fragments. Even though most epitopes in the literature have been defined using clade B reagents and the HIV_CONSV_ immunogen is an assemblage of HIV-1 clade A, B, C, and D consensus fragments, still 192 (71%) of these 270 HIV_CONSV_ epitopes are identical to an experimentally defined epitopes and additional 59 (22%) differ by only one aa, so that 251 (93%) epitopes differ by no more than a single-aa difference from a known epitope and thus may elicit a cross-reactive response.

### Vaccine construction and basic immunogenicity

**Figure 2:**
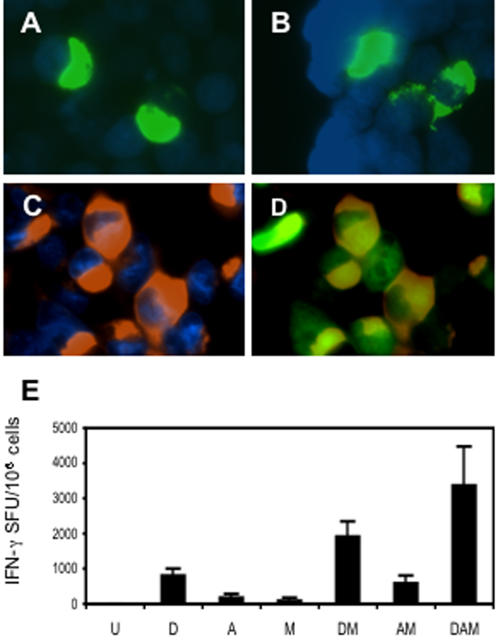
HIV_CONSV _protein expression in human cells and basic immunogenicity. A histochemical and DAPI staining of 293T cells transiently transfected with pTH.HIV_CONSV_ DNA (A), or infected with MVA.HIV_CONSV_ (B) or AdHu5.HIV_CONSV_ (C and D). HIV_CONSV_ protein expression was detected using mAb tag Pk at the C-terminus of the immunogen and a primary anti-Pk mAb followed by secondary FITC- (A and B) or AlexaFluor584- (C and D) conjugated detection antibodies. The AdHu5.HIV_CONSV _vaccine also expressed GFP, which co-localized with the HIV_CONSV_ expression (D). (E) BALB/c mice were immunized using the regimen indicated below, and the HIV_CONSV_-induced T cell responses were assessed in an ELISPOT assay using the H epitope. Results are shown as a mean±SD (n = 4). U–unimmunized; D–pTH.HIV_CONSV_ DNA; A–AdHu5.HIV_CONSV_; and M–MVA.HIV_CONSV_. For doses and timing, see Methods.

The *HIV_CONSV_* gene was made synthetically using ‘humanized’ aa codons[50] and its open-reading frame was preceded by a consensus Kozak sequence to -12 nucleotides[51] to maximize protein expression. A Met start codon was added to the first fragment. For initial studies, the gene was inserted into plasmid pTH DNA, human adenovirus serotype 5 (AdHu5) and modified vaccinia virus Ankara (MVA) vectors described previously[52] to yield pTH.HIV_CONSV_, AdHu5.HIV_CONSV_ and MVA.HIV_CONSV_ vaccines, respectively. The HIV_CONSV_ protein expression was demonstrated by immunofluorescence of transiently transfected or infected human embryonic kidney cells 293T using the mAb C-terminal tag (Fig. 2A–D). Using immunodominant epitope H, also known as P18-I10[49] and restricted by *H-2D^d^* and *L^d^*, the immunogenicities of individual vaccines and their heterologous prime-boost combinations were confirmed in the BALB/c mice. This demonstrated a strong priming with the pTH.HIV_CONSV_ DNA and ability to boost responses by heterologous vaccine vectors measured by *ex vivo* interferon (IFN)-γ ELISPOT (Fig. 2E) and ^51^Cr-release (not shown) assays. Particularly strong responses were elicited by the pTH DNA-rAdHu5-rMVA regimen (further designated DAM) delivering HIV_CONSV_ reaching a mean of 3375 spot-forming units (SFU)/10^6^ of freshly isolated splenocytes.

### Breadth of HIV_CONSV_ vaccine-induced immune responses in the BALB/c mice

**Figure 3:**
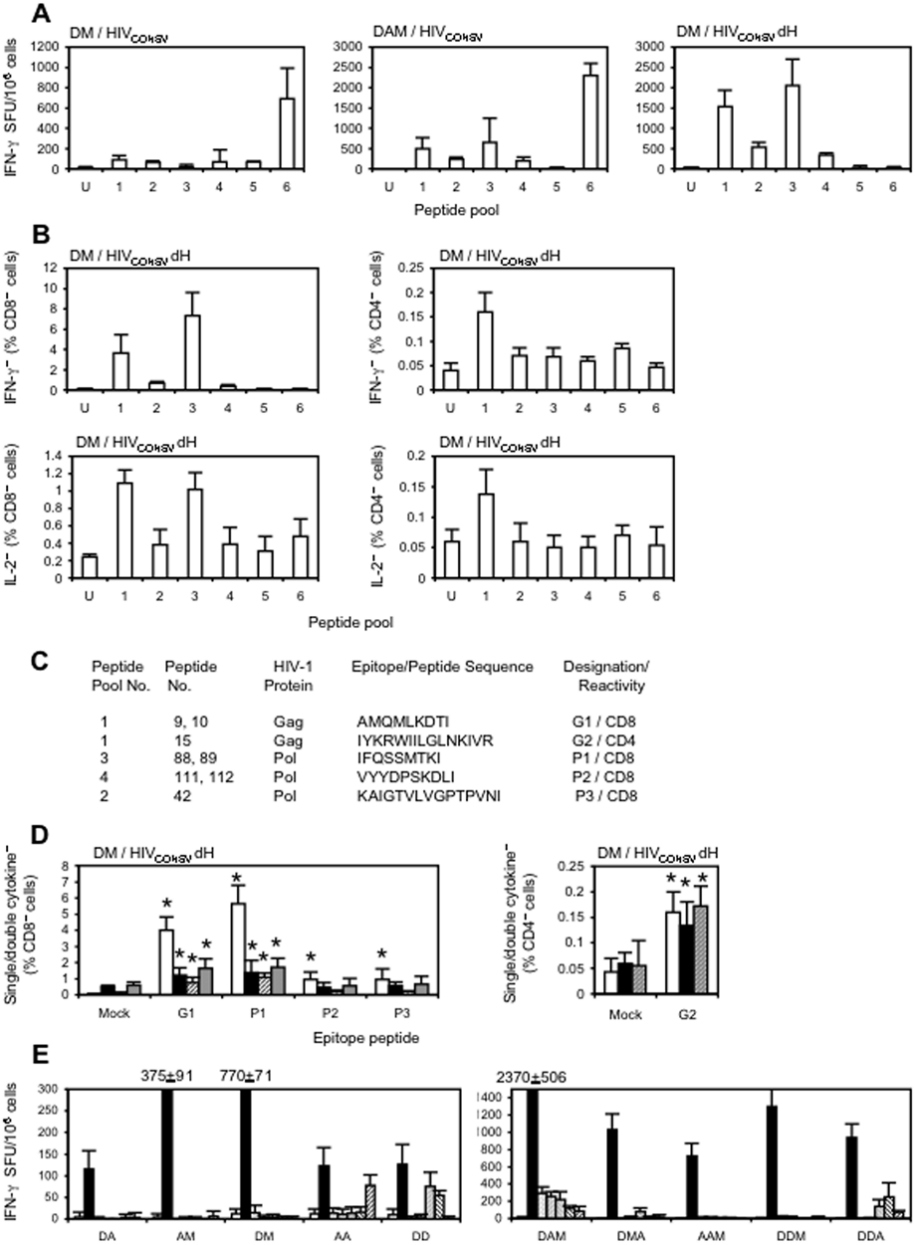
Breadth of HIV_CONSV_-induced T cell responses in BALB/c mice. Mice were immunized using the regimen and immunogen indicated above (A, B and C) or below (D) the graphs and the HIV_CONSV_-specific responses were determined in *ex vivo* ELISPOT (A and E) or ICS (B and D) assays detecting the indicated cytokines and using for restimulation overlapping peptide pools schematically shown in Fig. 1D (A and B) or individual epitope peptides (D and E). (C) Identified peptides or epitope sequences and their origin, name and T cell reactivity. In (D): white–IFN-γ; black–IL-2; stripy-IFN-γ+IL-2; and grey–TNF-α; *-responses significantly above the no-peptide background (p<0.05). In (E): white–no peptide followed from left to right by epitopes H, G1, G2, P1, P2 and P3. Results are shown as a mean±SD (n = 4). For doses and timing, see Methods.

The HIV_CONSV_ chimaeric protein is not a natural protein, and the new context resulting from concatenating the fragments may impact the processing of intact epitopes that are embedded within the fragments. It was therefore important to demonstrate that HIV_CONSV_ can induce T cell responses in mice and that epitopes recognized by human HLA-restricted T cells can be generated. We noted, as previously, that the response to the added H epitope dominated the T cell response in BALB/c mice (Fig. 3A). To avoid the H epitope domination of the T cell responses in the BALB/c mice[18], [53], vaccines expressing HIV_CONSV_dH immunogen with the H epitope deleted were constructed. Groups of BALB/c mice were immunized using the two strongest heterologous regimens from the previous experiment and the breadth of induced T cells was assessed using six pools of 32 peptides (15-mer overlapping by 11 amino-acid residues) corresponding to the whole HIV_CONSV_ protein (Fig. 1D). While weak responses were observed following the DM regimen, higher frequencies of T cells recognizing at least 5 peptide pools were elicited by the DAM regimen (Fig. 3A, left and middle panels). In both instances, responses to pools 1–4 were dominated by the H epitope in pool 6 and were much stronger when the HIV_CONSV_dH immunogen was used (Fig. 3A, right panel). Next, we showed that the DM regimen of the HIV_CONSV_dH vaccines induced both CD8^+^ and CD4^+^ T cells, which could produce IFN-γ and IL-2 in response to antigenic stimuli (Fig. 3B). Following identification of the individual pool peptides, minimal previously identified peptides were confirmed for some responses minimal epitopes[53] (Fig. 3C). Further analysis demonstrated elicitation of high quality T cells capable of production of IFN-γ, IL-2 and TNF-α (Fig. 3D) and killing of targets sensitized with MHC class I-restricted peptides (not shown). Finally, using the HIV_CONSV_ vaccines and the one CD4^+^ and five CD8^+^ T-cell epitopes, various dual and triple heterologous regimens were directly compared. This indicated that at the doses used, triple schedules were more immunogenic than the dual ones and indicated the superiority of DAM (Fig. 3E).

### HIV_CONSV_ vaccine-induced HLA-A2-restricted responses in transgenic mice

**Figure 4:**
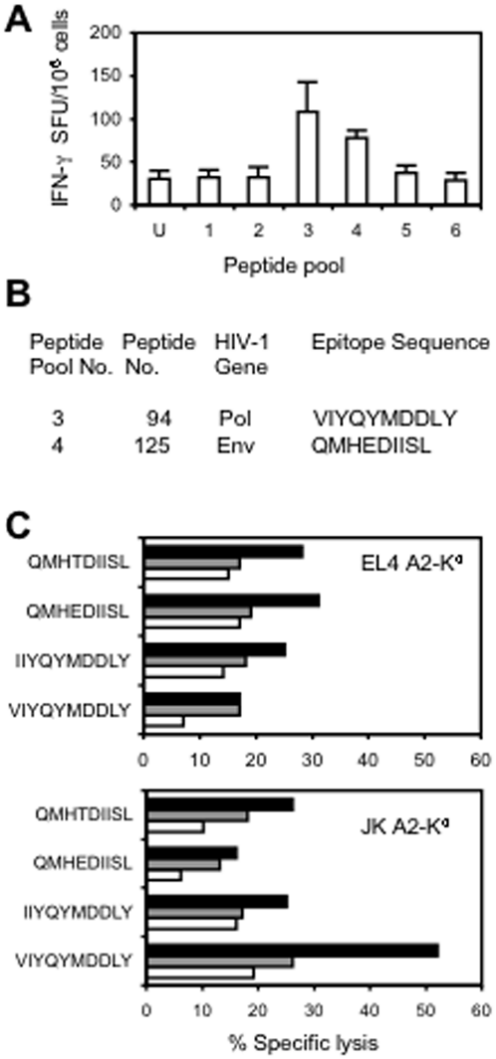
HIV_CONSV_-induced T cell responses in HLA-A*0201-transgenic mice, strain HHD. (A) Mice were immunized using the DAM regimen and the vaccine-induced responses were detected in an *ex vivo* ELISPOT assay. Results are shown as a mean±SD (n = 4). For doses and timing, see Methods. (B) Identified epitope peptides and their origin. (C) Killing of murine EL4 A2-K^d^ (top) and human JK A2-K^d^ (bottom) target cells sensitized with the shown peptides in a ^51^Cr-release assay after a 5-day *in vitro* peptide re-stimulation. Black, grey and white bars indicated effector to target ratios of 100, 50 and 25 to 1, respectively.

As a prelude to experiments in humans, we used genetically modified mouse strain HHD, which expresses as the only MHC class I molecule chimaeric human (α-1 and α-2) and mouse (α-3) HLA-A*0201 heavy chain covalently linked to the human β_2_m light chain. We used the most potent DAM regimen of the HIV_CONSV_ vaccines for induction of HLA-A*0201-restricted T cells and detected responses in *ex vivo* IFN-γ ELISPOT assay recognizing two peptide pools (Fig. 4A). Using the same assay, the fine specificities of these responses were mapped to two previously described[54], [55] although relatively uncommon, minimal epitopes (Fig. 4B), which were confirmed in a ^51^Cr-release assay on murine and human target cell lines expressing the HLA-A*0201 molecule. The epitopes were fully conserved in three out of the four consensus clade sequences, but the one aa substitution of the one outlier clade did not affect the killing (Fig. 4C). Note that the VIYQYMDDLY epitope encompasses the reverse transcription active site.

### Generation of HIV_CONSV_-specific responses in natural HIV-1 infection

**Figure 5:**
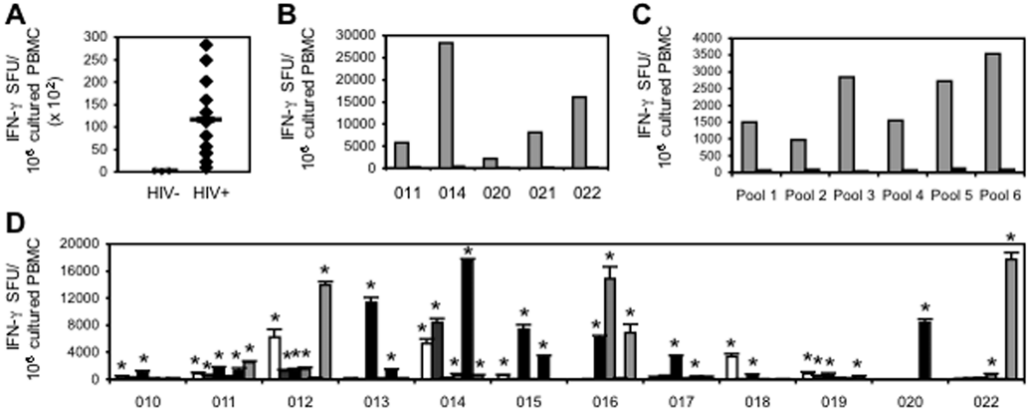
Recognition of HIV_CONSV_-derived peptides by PBMC from HIV-1-infected patients. The HIV_CONSV_-specific memory T cells were assessed in healthy and HIV-1-infected subjects using an IFN-γ ELISPOT assay after a 10-day peptide and cytokine culture. (A) Summed frequencies of HIV_CONSV_-specific cells detected in healthy (n = 9) and HIV-1-infected (n = 13) subjects. The bars show the group medians of 578 SFU/10^6^ and 8,092 SFU/10^6^ cells for the healthy and infected subjects, respectively. (B) In five subjects indicated below, cultured PBMC were left undepleted (grey) or depleted of CD8^+^ cells (black) prior to the ELISPOT assay. The difference between undepleted (median = 8,092 SFU/10^6^ cells) and CD8-depleted samples (median = 550 SFU/10^6^ cells) was statistically significant (*p* = 0.0313). (C) Responses to individual HIV_CONSV_-derived peptide pools as shown in Fig. 1D determined for the HIV-1-infected (grey) and healthy (black) subjects shown as medians. (D) Responses to individual peptides pools for each HIV-1-infected patient indicated below. Bars show a mean±SD of three assay wells and ‘*’ indicates a positive response according to criteria set in Methods. Due to sample shortage, subject 021 was not tested.

Next, we demonstrated that natural HIV-1 infection generally leads to generation of HIV_CONSV_-specific T cell responses, although these are usually smaller than the commonly seen immunodominant responses to epitopes in the more variable regions of HIV-1 and required in vitro expansions. All blood donors were tissue typed and where possible, the clade of their virus was identified (Table 1). In order to maximize sensitivity, PBMCs from infected patients were expanded in culture in the presence of HIV_CONSV_-derived peptide pools prior to the IFN-γ ELISPOT assay as described previously[31]. In this highly sensitive assay, 0 of 9 healthy HIV-1/2-uninfected lab volunteers had detectable responses to HIV_CONSV_ peptides, while 13 of 13 HIV-1-infected patients had HIV_CONSV_-specific memory T cells (Fig. 5A). In all five tested patients for whom we had sufficient frozen PBMC, depletion of CD8^+^ cells demonstrated that these responses were mediated by CD8^+^ T cells (Fig. 5B). These responses were broad and the median magnitude of all the HIV-1-infected patients for each peptide pool ranged between 1,000 and 3,500 SFU per 10^6^ cells in the cultured IFN-γ ELISPOT assay (Fig. 5C). With the exception of patient 020, twelve HIV-1-infected individuals responded to at least two peptide pools and 6 to three or more (Fig. 5D). Thus, conserved regions of the HIV-1 proteome included in the HIV_CONSV_ immunogen served as a source of T cell epitopes immunogenic during the course of natural HIV-1 infection.

**Table 1:** Tissue types and infecting viruses of human blood donors

Donor No.	HLA			HIV-1 Clade
	A*	B*	Cw*	
001^a^	1, 11	44, 51	7, 15	n.a.
002 a	2, 3	7, 13	6, 7	n.a.
003 a	1, 24	15, 18	3, 12	n.a.
004 a	1, 0201	7, 40	2, 7	n.a.
005 a	2, 3	7, 15	3, 7	n.a.
006 a	1, 0301	7, 08	7, 97	n.a.
007 a	2, 23/24	57, 42	2, 17	n.a.
008 a	1, 2	5001, 55	3, 6	n.a.
009 a	0201, 29	44, 13	6, 1601	n.a.
010 b	0102, 3303	44, 5802	n.d.	B/D
011 b	2, 29	45, 5802	6, 1601	D/A2 (CRF16)
012 b	2	15, 4402	3, 5	B
013 b	01, 11	18, 35	4, 7	B
014 b	24, 3401	40, 56	1, 4	B/C (CFR07)
015 c	3, 11	15, 4402	0303, 05	B
016 c	2601, 6802	70, 81	03, 04	n.d.
017 c	24	7, 18	7, 16	n.d.
018 c	1, 6801	5001, 1517	6, 7	n.d.
019 c	2601, 6802	70, 81	3, 4	n.d.
020 c	29, 32	7, 4401	1601	n.d.
021 c	0201, 0205	7, 18	n.d.	n.d.
022 c	03, 11	4201, 5301	4, 17	C

a HIV-1/2-uninfected subjects

b UK HIV-1-infected patients vaccinated with HIVA vaccines

c Patients infected with HIV-1 in Africa [4]

n.a. – not applicable; n.d. not done

## Discussion

A critical and contentious issue in HIV-1 vaccinology is how the vaccine-induced T cell responses will cope with both the intra- and inter-clade virus variabilities. Here, we describe a design and pre-clinical evaluation of vaccine immunogen HIV_CONSV_, which is based on the 14 most conserved regions of the HIV-1 proteome. These regions are well populated with known though less dominant CD8^+^ T cell epitopes, which are highly conserved. We demonstrated in BALB/c and HLA-A*0201-transgenic mouse strains that this vaccine immunogen can serve as a source of immunogenic epitopes. Furthermore, we detected HIV-1-specific memory T cells that could recognize HIV_CONSV_-derived peptides in 13/13 HIV-1-infected patients proving that HIV_CONSV_ -specific responses are commonly generated during natural HIV-1 infection.

Pathogens such as HIV-1 with highly variable genomes still have relatively conserved regions of the proteome, which are structurally or functionally important. Some of these regions might reflect a lack of a selective immunological pressure, i.e. absence of immunodominant or any epitopes that select out escape mutations, although one would expect to see some background synonymous and non-synonymous variability if there were no fitness cost to substitutions in such regions. Here, we confirm both in mice and humans that the conserved HIV_CONSV_ regions are not generally immunologically inert and contain T cell epitopes that are often recognized, although these tend to be less immunodominant than more variable epitopes in natural infection. This subdominance may be an advantage for the vaccine strategy, because it would allow elicitation of a T cell response that is different from that normally stimulated by HIV-1 (which fails to control the virus). The subdominance of many HIV_CONSV_ epitopes and influence of immunodominance was clearly demonstrated in the series of BALB/c mouse immunizations using the HIV_CONSV_ and HIV_CONSV_dH immunogens where a deletion of a single dominant ‘H’ epitope tripled the frequencies to several previously subdominant epitopes. Although 37 HLA-A2-restricted epitopes have been described, most patients only respond to the same two or three of these, which are immunodominant, but absent from the HIV_CONSV_ immunogen [56]. Therefore, it was encouraging that the HLA-A*0201-transgenic mice nevertheless generated responses to 2 conserved subdominant epitopes, one of which include the reverse transcriptase active site. It is noteworthy that responses to some subdominant epitopes have been shown to be more protective than that those recognizing their immunodominant companions [57]–[59]. In natural HIV-1 infection, many of the conserved epitopes present in this construct are subdominant as implied by the need for expansion of patients' PBMC prior to the response detection. In contrast the more variable escapable epitopes dominate to the extent that HLA type imprints changes in virus sequence in the patient populations [60], [61]. Our approach offers the possibility of changing the natural immunodominance by pre-infection vaccination, focusing the responses on highly conserved epitopes more like those seen by long term non-progressors with HLA B27 or B57 (ref. [62]).

In this work, we also directly compared various heterologous two- or three-component vaccination regimens and found that the most potent at the vaccine doses used was a combination of the three employed vectors in a DNA priming followed by sequential boosts with rHAdV-5 and rMVA, or the DAM regimen. Although this may depend of the doses used and be immunogen specific[63], triple prime-boost regimens were superior to the dual deliveries and merit testing in clinical trials.

A theoretical problem with this vaccine construct, a consequence of the chimaeric nature of the immunogen, is the possibility that unnatural stretches of aa at the boundaries of the fragments could also elicit T cell responses. In the work here, we did not identify any response to a junctional epitope, although we cannot exclude this happening with some HLA types and when the vaccine is tested in humans these responses should be sought to ensure there are no cases of immunodominance weakening the true anti-HIV-1 responses.

Although T cell epitopes in conserved regions of HIV-1 proteins were identified previously[64]–[72] and their value for vaccine development has been recognized[71], [72], the HIV_CONSV_ immunogen has a number of unique features. The HIV_CONSV_ immunogen is a chimaeric protein assembled from protein regions rather than epitopes, which enables broader coverage overlapping epitopes presented by multiple HLA proteins; it uses consensus sequences of the four major HIV-1 clades, which are in many segments identical to consensus sequences from multiple clades and this should allow a geographically broad deployment of the HIV_CONSV_ vaccines; it employs artificial clade consensus sequences designed to deal with the intra-clade variability; it combines sequences of the four clades sequentially rather than in parallel, which avoids epitope antagonism; and through the lower frequency of immunodominant epitopes, it favours induction of broader T cell responses. Our initial results indicate that human T cell responses to epitopes embedded in the chimaeric immunogen are common, although further study is needed to indicate how generalizable this is to other epitopes of HIV_CONSV_, and if the junction regions between fragments can stimulate problematic responses in humans. Finally, the theoretical global epitope coverage by the HIV_CONSV_ immunogen is narrower compared to the considerably more complex “mosaic” gene strategy, but the balance between optimizing epitope coverage and vaccine simplicity perhaps favours our HIV_CONSV_ approach.

The desirable, but not critical non-human primates studies with the HIV_CONSV_ immunogen are underway. Although the advantages and disadvantages of the HIV_CONSV_ vaccines or any of the other methods addressing the HIV-1 diversity discussed above can be argued theoretically and in model situations, whether or not any of them can induce broad enough anti-HIV-1 responses to decrease the transmission and/or reduce virus load in HIV-1-infected vaccine recipients will be only proven in clinical trials.
