# Correction: Design and Pre-Clinical Evaluation of a Universal HIV-1 Vaccine

**DOI:** 10.1371/annotation/fca26a4f-42c1-4772-a19e-aa9d96c4eeb2

**Published:** 2011-03-08

**Authors:** Sven Létourneau, Eung-Jun Im, Tumelo Mashishi, Choechoe Brereton, Anne Bridgeman, Hongbing Yang, Lucy Dorrell, Tao Dong, Bette Korber, Andrew J. McMichael, Tomáš Hanke

The authors would like to add the following to the funding information: "The study was supported by funding from the NIHR Oxford Biomedical Research Centre programme."

